# Comparing metacognitive monitoring between native and non-native speaking primary school students

**DOI:** 10.1007/s11409-021-09261-z

**Published:** 2021-04-14

**Authors:** Florian J. Buehler, Mariëtte H. van Loon, Natalie S. Bayard, Martina Steiner, Claudia M. Roebers

**Affiliations:** Institute of Psychology, Department for Developmental Psychology, University of Bern, Fabrikstrasse 8, 3012 Bern, Switzerland

**Keywords:** Metacognition, Monitoring, Language, Non-native speakers, Paired-associates task, Text comprehension

## Abstract

Metacognitive monitoring is a significant predictor of academic achievement and is assumed to be related to language competencies. Hence, it may explain academic performance differences between native and non-native speaking students. We compared metacognitive monitoring (in terms of resolution) between native and non-native speaking fourth graders (~ 10 year olds) in two studies. In Study 1, we matched 30 native and 30 non-native speakers and assessed their monitoring in the context of a paired-associates task, including a recognition test and confidence judgements. Study 1 revealed that recognition and monitoring did not differ between native and non-native speaking children. In Study 2, we matched 36 native and 36 non-native speakers and assessed their monitoring with the same paired-associates task. Additionally, we included a text comprehension task with open-ended questions and confidence judgments. We replicated the findings of Study 1, suggesting that recognition and monitoring do not necessarily differ between native and non-native speakers. However, native speaking students answered more open-ended questions correctly than non-native speaking students did. Nevertheless, the two groups did not differ in monitoring their answers to open-ended questions. Our results indicate that native and non-native speaking children may monitor their metacognitive resolution equally, independent of task performance and characteristics. In conclusion, metacognitive monitoring deficits may not be the primary source of the academic performance differences between native and non-native speaking students.

Can you imagine following a mathematics class in a foreign language when you were as young as ten years old? Across the world, many non-native speaking schoolchildren face such challenges every day. They get instructions in a language that they do not speak at home. Compared to their native speaking peers, this is likely to be an extra challenge for their learning. Not surprisingly, international studies show that non-native speaking children typically underperform in school subjects, such as reading, mathematics, and science (OECD 2012, 2018). Although non-native speaking children build a substantial and growing population in countries of the Organisation for Economic Cooperation and Development (OECD 2019), only very little is known about the mechanisms underlying their often observed underachievement. In this contribution, we focus on one consistent predictor of school achievement in primary school children, which is metacognitive monitoring (Freeman et al. 2017; Roebers et al. 2014), describing the ability to evaluate one’s ongoing cognitive processes (Nelson and Narens 1990; Schneider and Löffler 2016). From a theoretical perspective, monitoring is likely related to language competencies (Ebert 2015). Therefore, we aim to explore how language competencies are related to children’s monitoring and whether monitoring differences between native and non-native speaking students may contribute to performance differences in a learning task.

Large-scale assessments such as The Programme for International Student Assessment (PISA) conducted by the OECD reveal that speaking the language of instruction at home is related to the prospect of reaching the baseline level of proficiency in the three main PISA subjects: reading, mathematics, and science (OECD 2012, 2018). The largest differences in favour of native speaking children are typically reported in reading performance. Unlike non-native speakers, native speaking children hear and speak the language of instruction at home, from which their language skills are likely to benefit. Indeed, speaking the language of instruction at home is strongly related to reading performance (OECD 2012). In other words, children’s linguistic environment at home seems to be vital for academic achievement and, hence, non-native speaking children may be disadvantaged in school. Towards the end of primary education, aspects of self-regulated learning, such as metacognitive monitoring skills, become increasingly important and may contribute to achievement gaps between native and non-native speaking students.

Metacognition consists of declarative (knowledge about the importance of person, task, and strategy variables for cognition; Flavell and Wellman, 1977) and procedural aspects (monitoring and regulation of memory processes; Schneider and Löffler 2016). Declarative metacognition and self-regulated learning strategies are related to academic achievement in school aged children (Artelt et al. 2001; Schneider and Artelt 2010; Veenman et al. 2005). Procedural metacognitive abilities were consistently found to be related to academic achievement in primary and secondary school children (Dunlosky and Metcalfe 2009; Freeman et al. 2017; Kleitman and Gibson 2011; Roebers et al. 2014; Stankov et al. 2012; Stankov et al. 2014). In a very recent study, second and fourth graders’ inaccurate metacognitive monitoring played a key role in understanding ineffective self-regulated learning strategies (Bayard et al. 2021). One can therefore assume that test performance in a learning task is directly related to metacognitive monitoring and control processes in primary school children (Roebers et al. 2014). Against this background, we hypothesize that differences in monitoring abilities contribute to non-native speaking children’s underperformance.

Monitoring is typically assessed by asking individuals to give confidence judgments concerning their answers and relating these to actual task performance (Dunlosky et al. 2016). Within the literature, there are different approaches to quantify monitoring. The present contribution focuses on two complement measures of monitoring resolution, targeting the ability to metacognitively distinguish between correct and incorrect answers across items (Dunlosky et al. 2016; Dunlosky and Thiede 2013). This implies that an individual gives higher confidence judgments for answers that turn out to be correct than for those answers that turn out to be incorrect. Especially in educational and developmental contexts, monitoring resolution measures are considered to provide the most valuable insights into children’s challenges when monitoring (for a review see Roebers 2017; Schneider and Löffler 2016).

Crucial for non-native speakers is the fact that metacognitive monitoring is typically assessed verbally (“*How sure are you that you answered this question correctly?*”). This is of high relevance as language is probably an essential variable in developing children’s knowledge about mental processes (Ebert 2015). It provides a means to think, talk, and learn about mental states and processes (Astington and Baird 2005; Harris et al. 2005). Ebert (2015) outlined several theoretical reasons why language features might be associated with metacognition. A grammatical understanding may support children to represent mental states. Moreover, acquiring mental words may also foster a conceptual understanding, facilitating learning about unobservable cognitive processes, such as metacognitive monitoring. Finally, language skills may facilitate verbal interactions with other individuals and foster learning about the mental world, including metacognition. Against this theoretical background, it is not surprising that early language competencies were related to later metacognition (Annevirta et al. 2007; Ebert 2015; Lecce et al. 2010; Lockl and Schneider 2007). In conclusion, metacognitive abilities might vary across individuals with different language backgrounds and skills, such as native and non-native speaking children.

In contrast, research assessing *multilingual*s suggests an advantage for multilinguals in higher order cognitions. Meta-analyses found advantages for bilinguals in metalinguistic and metacognitive awareness, working memory, abstract and symbolic representation, attentional control, and problem solving (Adesope et al. 2010; Grundy and Timmer 2017). Interestingly, bilinguals outperformed monolinguals in verbal and non-verbal executive function tasks, suggesting a general bilingual advantage in working memory tasks (Grundy and Timmer 2017). Note that bilinguals in those studies were identified as being equally (or almost equally) proficient in two languages (Adesope et al. 2010; Grundy and Timmer 2017). In comparison, non-native speakers are second language learners in the language of instruction. It may be that speaking various languages is potentially beneficial for higher order cognition, but this is likely the case *only* when one master those languages on a proficient level. Therefore, it remains unclear whether the bilingual advantage in higher order cognitions applies to non-native speakers.

## The present study

The present study focuses on metacognitive monitoring in native and non-native speaking 4^th^ graders. This age range appears especially important as children soon face the transition into secondary education. We assessed the participants with a paired-associates task. With this task, we are avoiding effects of prior knowledge (cf. Destan et al. 2014; Roderer and Roebers 2010), and we can surely expect a sufficiently developed ability to metacognitive discriminate between likely correct and potentially incorrect responses. This also brings about the advantage that the participants are free to remember the content (pictures) in any language. Therefore, instruction’s language should not be of high relevance for first-order performance (recognition) in the paired-associates task. Based on international assessments investigating academic achievement (OECD 2012, 2018), we would expect first-order performance differences in favour of native speaking students, but due to the paired-associates task characteristic’s such differences should not be pronounced. The scarce literature does not allow us to predict whether native and non-native speakers differ in metacognitive monitoring. Language abilities and monitoring are likely related (Ebert 2015). Based on the findings that non-native speakers are disadvantaged in language competencies (OECD 2012, 2018), one can expect that non-native speaking children’s monitoring skills are inferior compared to native speaking children. The bilingual advantage claims that speaking multiple languages is beneficial for higher order cognitions, such as metalinguistic and metacognitive awareness (Adesope et al. 2010; Grundy and Timmer 2017). However, -other than bilinguals- non-native speakers are not proficient in multiple languages. Finally, the specific link between abilities in the language of instruction and monitoring was not yet investigated. We took an explorative approach to examine whether native and non-native speaking children differ in their monitoring abilities -over and above the to-be-expected performance differences. A better understanding of underlying mechanisms of monitoring might contribute to a better understanding of disadvantaged students, such as non-native speaking children (Freeman et al. 2017; Kleitman and Gibson 2011; OECD 2018; Roebers et al. 2014; Stankov et al. 2012, 2014).

## Method Study 1

### Participants

Participants were 133 4^th^ grade children. We recruited them from public schools in the vicinity of a mid-sized university town. Parents had signed informed consent, and children gave consent verbally before testing. Based on teachers’ information, we excluded four participants with pathologies such as autism spectrum disorder or ADHD. Furthermore, we excluded three children due to technical issues and one child that broke off the task. Finally, we excluded three participants due to ceiling effects (recognition score = 100%) and two participants due to floor effects (recognition score at chance level ≤ 25%) in the recognition task.

To build groups of native and non-native speaking children, we asked teachers to indicate each student’s mother tongue(s). Teachers retrieved such information from official documents, including demographic information about their students or by asking the students themselves. Our remaining sample consisted of 68 native speaking students, 34 non-native speaking students, and 18 multilinguals (i.e., children who speak more than one language on a native level). Multilingual children cannot be allocated to one of the two groups, as it remains unclear whether their abilities in the language of instruction match the native or the non-native speaking group. Furthermore, the small number of multilinguals did not allow further analyses; hence, we excluded them from our analyses (*n* = 18). To ensure comparability of the two differently sized groups (68 native speakers vs. 34 non-native speakers), we matched each non-native speaking student with a native speaking peer. Non-native speaking students were individually matched to native speaking students by age (tolerance = 3 months) and gender. We could not match four non-native speaking children as their age exceeded that of any native speaking peer, therefore we excluded them. The matching led to two comparable (considering age and gender) and equally sized groups of native (*n* = 30; *M*_*age*_ = 10.79y; *SD*_*age*_ = 5.73m; 47% girls) and non-native speaking (*n* = 30; *M*_*age*_ = 10.76y; *SD*_*age*_ = 5.62m; 47% girls) participants. All native speaking children spoke German as their mother tongue. The mother tongues of the non-native speakers are indicated in Table 1.

**Table 1:** Mother tongue of non-native speaking children, Study 1

Language	*N*	%
Albanian	10	33.30
Kurdish	4	13.30
Serbian	2	6.70
Somali	2	6.70
Turkish	2	6.70
African Language (unknown)	1	3.30
Arabic	1	3.30
Croatian	1	3.30
Farsi	1	3.30
French	1	3.30
Hungarian	1	3.30
Polish	1	3.30
Portuguese	1	3.30
Tamil	1	3.30
Tigrinya	1	3.30
Total	30	100

*Note.* Teachers were asked to indicate the mother tongue of their students

### Procedure and materials

**Fig. 1:**
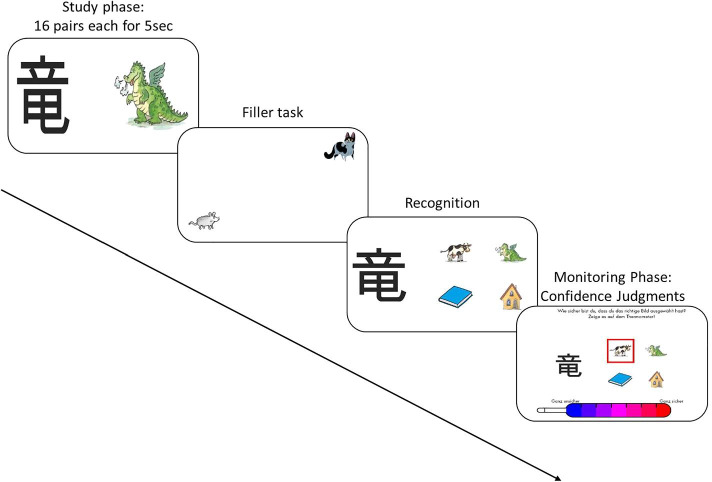
Procedure of the paired-associates task: After studying the 16 Kanji-picture pairs, children had to recognize for each Kanji the correct picture out of 4 options and provide confidence judgments

We conducted the study following the declaration of Helsinki. The local ethics committee (Faculty of Humanities of the University of Bern; approval number: 2016-08-00004) approved the study’s procedure. We conducted a group assessment in the usual classroom setting. Two trained investigators supervised children within a class. The task was presented on a tablet computer (11.6″) with a touch screen. We gave general instructions in German at the start. During the task, further instructions were given orally via headphones and visually as text on the screen (both in German). Before starting the task, children completed a practice trial to familiarize themselves with the material and the test format. The task was organized in 3 phases: The study phase, recognition, and monitoring phase (Fig. 1). The task lasted approximately 30 min.

#### Study phase (Kanjis)

In the study phase, the subjects were told to remember 16 pairs of pictures and that they will be asked to recognize those pairs later in the task. The pairs were presented in random order and composed of a Kanji (a Japanese character) and its depicted meaning (a colour drawing). Each pair appeared for 5 sec. After the study phase, subjects conducted a filler task (1 min.) to prevent rehearsal and other memory strategies. The filler task consisted of an easy mouse-catching game on the tablet. The children steered a cat with one finger and tried to catch a mouse.

We piloted a large pool of item pairs beforehand to ensure sufficient variability concerning item difficulty. We included pairs with a difficulty index between 0.11 and 0.78 in the present study (Moosbrugger and Kelava 2008). The identical task was applied in previous studies (Destan et al., 2014; Destan & Roebers, 2015).  

#### Recognition (Kanjis)

In the recognition test, one Kanji at a time was depicted with four alternative drawings. Children were instructed to select the best alternative of the four by touching the respective drawing. All alternatives had been presented during the study time and were thus familiar to the children. A forced report selection characterized the recognition phase. When children were unsure about the correct answer, they were still asked to choose one of the four pictures. After selecting, a red frame surrounded the selected drawing. No feedback about their recognition performance was provided.

#### Recognition monitoring

Immediately after selecting an alternative in the recognition test, the monitoring judgment (confidence judgment) for this particular trial was collected. Children were asked: “*How sure are you that you have chosen the correct answer*?” They had to indicate their confidence on a 7-point Likert scale, presented as a thermometer, by touching the thermometer’s respective colour with their index finger. The thermometer ranged from blue (indicating “unsure”) to red (indicating “very sure”; adapted from Koriat and Shitzer-Reichert 2002). Children received detailed instructions, and they practiced with items before starting with the task.

### Measures

For recognition, we computed the mean percentage of correctly recognized Kanjis out of the 16 to-be-remembered pairs for each participant. For metacognitive monitoring, we coded the thermometer’s confidence judgments as values ranging from 1 (very unsure) to 7 (very sure). There are many monitoring measures, and each has its strengths and weaknesses. We were specifically interested in a child-appropriate and classical monitoring measure that relates confidence judgments to item level performance, as research shows that primary school children, especially progress in their monitoring of incorrect answers (Howie and Roebers 2007; Roebers et al. 2007). Thus, we focused on two resolution measures, which allow to take a differentiated perspective on confidence judgments, by contrasting judgments concerning correct and incorrect answers: (a) metacognitive discrimination primarily targeting children’s growing ability to experience and report different degrees of confidence on the continuum of confident-unconfident; (b) intra-individual Gamma correlation between recognition accuracy and the reported confidence on item level. Besides the slightly different perspective on participants monitoring resolution, the inclusion of multiple measures enables to evaluate whether they converge on the same qualitative outcomes (Dunlosky et al. 2016; Dunlosky and Thiede 2013; Murayama et al. 2014).

For the discrimination score, we subtracted mean confidence judgments for incorrectly recognized Kanjis from mean confidence judgments for correctly recognized Kanjis (cf. Dunlosky and Thiede 2013; Roebers 2002). Positive discrimination values indicate that children were reliably more confident when their answer was correct than when it was incorrect and could experience different degrees of confidence. We also computed intra-individual Gamma correlations (Nelson 1984) between confidence judgements and recognition (correct vs. incorrect). Gamma correlations closer to 1 indicate a more proficient monitoring resolution, whereas values closer to 0 indicate lower monitoring resolution. Although Gamma correlations are the most frequently reported measure in metacognitive research, including students and adults (Dunlosky et al. 2016), they bear some disadvantages when used for children’s data (see Roebers and Spiess 2017).

### Analyses

We conducted a multivariate ANOVA with mother tongue (native vs. non-native speaking) as a grouping variable and recognition, the monitoring discrimination score, and Gamma correlations as dependent variables to test for group differences. We included all variables in one model to control for multiple comparisons issues.

## Results Study 1

Means for native and non-native speaking subjects are displayed in Table 2. Descriptive statistics revealed that native and non-native speaking students recognized a similar amount of Kanjis correctly. Both native, *F*(1, 29) = 78.76; *p* < *.*01; ${\upeta }_{p}^{2}$ = 0.73, and non-native speakers, *F*(1, 29) = 37.84; *p* < *.*01; ${\upeta }_{p}^{2}$ = 0.57, were more confident in correct recognitions compared to incorrect recognitions. Moreover, Gamma correlations between confidence judgments and recognition performance were significantly different from zero in both groups (Table 2).

**Table 2:** Means of performance and monitoring measures in Study 1 and Study 2 (SD in parentheses)

	Performance [%]	CJ correct response	CJ incorrect response	Monitoring Discrimination	Gammas
**Study 1** Paired-associates task					
Native speaking	60.21 (16.04)	5.20 (0.92)	3.72 (1.40)	1.49 (0.92)*	0.60 (0.43)**
Non-native speaking	58.33 (15.69)	5.29 (0.94)	4.23 (1.42)	1.06 (0.94)*	0.45 (0.38)**
**Study 2** Paired-associates task					
Native speaking	53.99 (16.17)	5.19 (1.03)	4.00 (1.30)	1.19 (0.90)*	0.50 (0.30)**
Non-native speaking	53.30 (14.21)	5.54 (0.81)	4.53 (1.37)	1.00 (1.10)*	0.40 (0.46)**
**Study 2** Text comprehension task					
Native speaking	${54.40 (17.76)}^{+}$	5.27 (1.13)	4.23 (1.30)	1.05 (1.17)*	0.46 (0.50)**
Non-native speaking	38.43 (22.21)	5.06 (1.43)	3.78 (1.17)	1.29 (1.50)*	0.54 (0.47)**

*Note.* CJs were indicated on a 7-point Likert scale. CJ = Confidence Judgments, Monitoring Discrimination = CJ correct recognition – CJ incorrect recognition, Gammas = Intra-individual correlations between task performance and confidence judgments; *p < .01 native and non-.native speaking participants gave significantly higher CJs when their response was correct vs. incorrect; **p < *.*001 all Gammas were significantly different from zero;${}^{+}$ *p* < .01 Native speakers answered significantly more open questions correctly than non-native speakers, in Study 2

A multivariate ANOVA including recognition, monitoring discrimination scores, and Gamma correlations as dependent variables with use of Pillai’s trace did not show significant group differences between native and non-native speaking students, *F*(3, 56) = 1.05; *p* = *.*38; ${\upeta }_{p}^{2}$ = 0.05. In sum, native and non-native speaking children did not differ significantly in recognition performance or monitoring resolution in the paired-associates task.

## Discussion Study 1

In Study 1, we investigated metacognitive monitoring of native and non-native speaking 4^th^ graders in a paired-associates task (Kanjis). Based on the scarce literature, we took an explorative approach. Results revealed that native and non-native speaking subjects did not differ in the number of correctly recognized Kanjis. Based on confidence judgements, we computed discrimination scores and Gamma correlations as measures of monitoring resolution. Native and non-native speaking children adequately discriminated between correctly and incorrectly answered items, as indicated by both the discrimination scores and Gamma correlations. Most importantly, we did not find any differences between native and non-native speaking children in either of the two monitoring resolution measures. In other words, native and non-native speaking children monitored their recognition in the Kanji task equally well.

Unlike PISA studies, we did not find performance disadvantages for non-native speakers (OECD 2012, 2018). We assessed subjects with a paired-associates task, which may be considered a language-reduced task, as the material was presented in the form of images. Thus, recognition performance might be independent of children’s competencies in the language of instruction. As recognition performance was comparable between the two groups, the included monitoring measures are likely to have estimated children’s monitoring skills about equally accurate (cf. Galvin et al. 2003; Maniscalco and Lau 2012; Roebers and Spiess 2017). These aspects together might explain why we did not find any differences between language groups. Regarding metacognitive monitoring, our findings may indicate that advantages in higher order cognitions do not occur simply through exposure to multiple languages and, thus, do not necessarily emerge when comparing native with non-native speakers. The level of mastery of those languages may be crucial for benefits in higher order cognitions. Therefore, it may be that only those who speak various languages at a proficient level –such as true bilinguals- benefit. Based on the assumption that native and non-native speakers differ in language competences, and based on previous research suggesting a theoretical and an empirical link between metacognition and language abilities (Annevirta et al. 2007; Ebert 2015; Lecce et al. 2010; Lockl and Schneider 2007), our results warrants replication in a more language-related task.

Therefore, we conducted a second study with an independent sample, for which we assessed children’s monitoring resolution with the same paired-associates task (Kanjis) as in Study 1 and a text comprehension task. This allowed estimating the influence of a language-based task on non-native speaking children’s monitoring. In contrast, to study 1, we assessed participants’ abilities in the language of instruction to evaluate individual differences in language competencies between native and non-native speaking subjects. For the Kanji task, we expected to replicate the findings of Study 1, such that native and non-native speaking children would not differ in recognition performance and metacognitive monitoring resolution. We expected that native speaking students outperform non-native speaking students for the text comprehension task, as performance differences between native and non-native speakers are typically visible in language-related tasks (OECD 2012). Regarding metacognitive monitoring, the text comprehension task’s high linguistic demands may impair monitoring abilities of non-native speakers. However, it remains unclear whether monitoring competencies are affected by language abilities. Thus and again, we took an explorative approach for Study 2.

## Method Study 2

We draw the sample of Study 2 from a larger research project on children’s developing metacognitive skills. Selected aspects of children’s metacognitive development have been reported previously, such as recognition performance, confidence judgements (gamma correlations) and response latency (time taken for recognition and confidence judgments in ms) for the Kanjis task and open question performance and confidence judgements (discrimination scores and gamma correlations) for the text comprehension task (Roebers et al., 2019; Steiner et al., 2020). However, non-native speaking children were excluded in these previous reports. This manuscript’s unique contribution is the focus on non-native children’s monitoring, including a comparison with a subsample of native children from previous reports.

### Participants

For Study 2, 151 4^th^ graders participated. We recruited the children from public schools in the vicinity of a mid-sized university town. Parents had signed informed consent, and children gave verbal consent before testing. We excluded two participants with pathologies such as ADHD, relying on the teacher’s information. As in Study 1, we excluded eight participants due to ceiling effects (recognition score = 100%) and one participant due to floor effects (recognition score at chance level ≤ 25%) in the recognition task, and 13 participants due to floor effects (no open question answered correctly) in the text comprehension task.

To build groups of native and non-native speaking children, we asked teachers to indicate each student’s mother tongue(s). Teachers retrieved such information from official documents, including demographic information about their students or by asking the students themselves. Our sample consisted of 78 native speaking students, 38 non-native speaking students, and ten multilinguals. Multilingual children cannot be allocated to one of the two groups, as it remains unclear whether their abilities in the language of instruction match the native or the non-native speaking group. The small number of multilinguals does not allow further analyses; hence, we excluded them from our analyses (*n* = 10). To ensure comparability of the two differently sized groups (78 native speakers vs. 38 non-native speakers), we matched each non-native speaking student with a native speaking peer. Matching was identical to Study 1. We could not match two non-native speaking children as their age exceeded that of any native speaking peer, therefore excluding them. The matching led to two comparable (considering age and gender) and equally sized groups of native (*n* = 36; *M*_*age*_ = 10.10y; *SD*_*age*_ = 3.70m; 44% girls) and non-native speaking (*n* = 36; *M*_*age*_ = 10.14y; *SD*_*age*_ = 4.26m; 44% girls) participants.

Furthermore, we asked teachers to rate participants’ language competencies in school instruction language on a scale from 1 (below average) to 5 (very good). On average, teachers rated the language competences of their native speaking students (*M* = 3.58; *SD* = 1.08) higher than the language competences of their non-native speaking students (*M* = 3.06; *SD* = 1.17), *t*(70) = 1.99; *p* = 0.05. All native speaking children spoke German as their mother tongue. The mother tongues of the non-native speakers are indicated in Table 3.

**Table 3:** Mother tongue of non-native speaking children, Study 2

Language	*N*	%
Albanian	11	30.56
Italian	6	16.67
Tamil	3	8.33
French	3	8.33
Hungarian	3	8.33
Serbian	2	5.56
Spanish	2	5.56
Portuguese	2	5.56
Arabic	1	2.78
Croatian	1	2.78
Turkish	1	2.78
Urdu	1	2.78
Total	36	100

*Note*. Teachers were asked to indicate the mother tongue of their students

### Procedure and materials

Participants completed a paired-associates task (Kanjis identical to Study 1) and a text comprehension task. We conducted a group assessment in the usual classroom setting of the children. For testing, we split the classes into groups of 6 to 11 children, and two trained investigators supervised each group. One group started with the paired-associates task, whereas the other group started with the text comprehension task. We counterbalanced the task order. We gave general instructions orally in German at the start. Further instructions during the task were given orally via headphones and visually as text on the screen. The paired-associates task and the text comprehension task lasted approximately 30 min each. The materials and procedure of the paired-associates task were identical to Study 1 and are presented in Fig. 1.

#### Text comprehension task

**Fig. 2:**
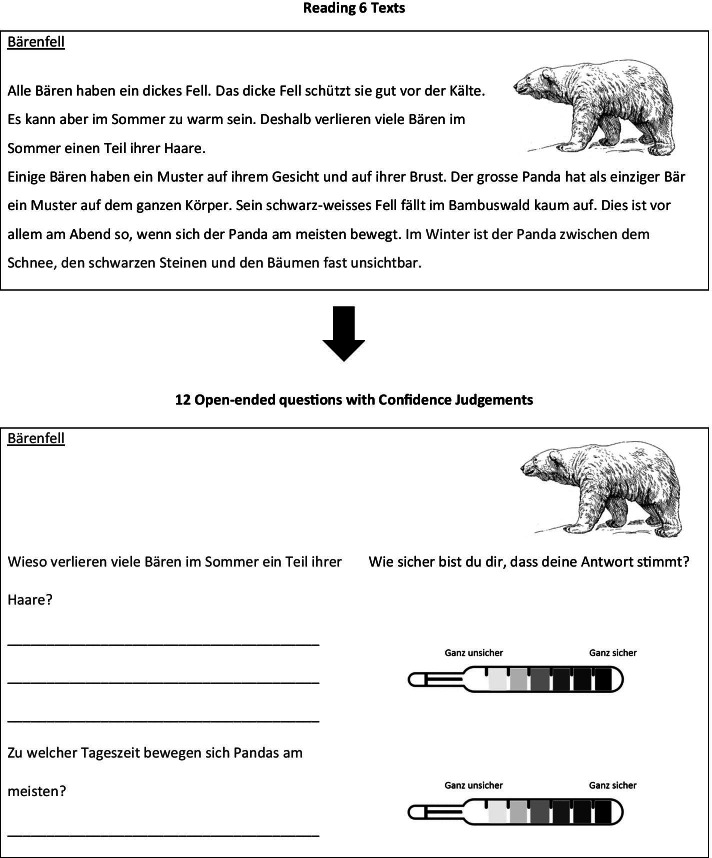
Procedure of the text comprehension task: After reading each text, children had to answer open questions and provide confidence judgements. Figure adapted from Steiner et al. (2020)

We gave general instructions orally in German at the start. During the task, the participants could read the instructions (in German), and they were repeated individually if needed. The general instructions included the nature of all upcoming tests. The text comprehension task included 3 phases: a study phase (text reading), answering open-ended questions about the read texts, and a monitoring phase (Fig. 2). Details about the task are reported by Steiner et al., (2020).

##### Study phase (Texts)

Students had to read six expository texts in German on a tablet (11.6″). Children could not move forward or backward between the texts. Study time was self-paced. However, the minimum reading duration was 10 sec per text. The text font was Futura Std. Books and the size was 25 pcts. Topics were animals (Bees, Bears, Dragonflies, and Camels), geographical subjects (Tropics, Desert, Egypt, Nile, Seasons, and Stars), or physiological processes (Catching a Cold, Chewing gum). Participants received the texts in random order.

We conducted a pilot study for choosing the texts and the open-ended questions for the present study. We translated and adapted the texts from previous studies (De Bruin et al. 2011; van Loon et al. 2015). We chose text-question sets that resulted in a similar amount of easy (~ 30%), medium (~ 40%), and difficult (~ 30%) open-ended questions. The mean length of the chosen texts was 126 words. The text’s complexity was 37.81 LIX (readability index; see Björnsson 1983), indicating that readability ranged between easy and moderate.

##### Open-ended questions

The text comprehension test consisted of 12 open-ended questions (2 questions per text) presented in a booklet as a paper–pencil test. Answers to open-ended questions could range from a single word to a full sentence (cf. Magliano et al. 2007). Hence, we included two kinds of open-ended questions to represent that question format fully. For each text, one of the open-ended questions required a single word (“At what time of the day are Pandas the most active?”) and the other a sentence (“Why do bear loose hair during summer?”). Participants were encouraged to answer all open-ended questions. However, when they could not think of any answer, they could put a question mark instead.

##### Monitoring (of answers)

Immediately after answering each open-ended question, children had to rate their confidence that the answer was correct. Specifically, children were asked: “*How sure are you that your answer is correct*?” For that purpose –as in the paired-associates task- a 7-point Likert scale was presented to the right of each question. The same thermometer scale was depicted, ranging from very “unsure” to “very sure”.

### Measures

We used identical measures for the paired-associates task as in Study 1. We computed recognition scores and two different monitoring resolution measures a discrimination score (difference in confidence between correctly and incorrectly recognized items) and intra-individual Gamma correlations between confidence judgments and recognition (see above).

#### Text comprehension performance

We coded answers to the open-ended questions as true (1) or false (0). In line with van Loon et al. (2015), we emphasized comprehension during scoring. Thus, we scored verbatim responses as well as gist responses as correct. Two independent raters coded all answers. Interrater reliability was very high (*κ* = 0.93, *p* < 0.001).

We coded question marks as omissions (Roebers et al. 2007). Native speaking subjects omitted 12.27% (*SD* = 12.20) and non-native speaking students 14.58% (*SD* = 14) of their answers, respectively. There was no missing data in the native speaking students and very little missing data in the non-native speaking students (*M* = 0.23; *SD* = 1.39). In the analyses, we included only completed test items because participants did not give confidence judgements if they had not come up with an answer. For further analyses, we computed the percentage of correct answers out of all answered open-ended questions.

#### Text comprehension monitoring

We coded the thermometer’s confidence judgments as values ranging from 1 (very unsure) to 7 (very sure). To assess text comprehension monitoring, we computed the same monitoring resolution measures as for the paired-associates task. Specifically, we subtracted mean confidence judgments for incorrectly answered open-ended questions from mean confidence judgments for correctly answered open-ended questions for a discrimination score (Dunlosky and Thiede 2013; Roebers 2002). Moreover, we computed intra-individual Gamma correlations (Nelson 1984) between confidence judgments and text comprehension (correct vs. incorrect).

### Analyses

We conducted separate but identical analyses for the paired-associates task (identical analyses as in Study 1) and the text comprehension task. We conducted a multivariate ANOVA with mother tongue (native vs. non-native speaking) to test for group differences for the text comprehension task as a grouping variable and text comprehension, monitoring discrimination scores, and Gamma correlations as dependent variables. We included all variables in one model to control for multiple comparisons problems.

## Results Study 2

Means for native and non-native speaking participants are displayed in Table 2. Descriptive statistics for the paired-associates task revealed that native and non-native speaking students recognized a similar amount of Kanjis correctly. Both native, *F*(1, 35) = 63.12; *p* < 0.01; ${\upeta }_{p}^{2}$ = 0.64, and non-native speakers *F*(1, 35) = 29.75; *p* < 0.01; ${\upeta }_{p}^{2}$ = 0.46, were more confident in their recognition when their answers were correct compared to incorrect recognitions. Moreover, Gamma correlations between confidence judgments and recognition performance were substantially different from zero in both groups (Table 2).

Descriptive statistics for the text comprehension task revealed that native speakers correctly answered more open-ended questions than non-native speakers. However, native, *F*(1, 35) = 28.98; *p* < 0.01; ${\upeta }_{p}^{2}$ = 0.45, and non-native speakers, *F*(1, 35) = 26.36; *p* < 0.01; ${\upeta }_{p}^{2}$ = 0.43, were more confident in their responses to the open-ended questions when their answers were correct compared to incorrect answers. Moreover, Gamma correlations between confidence judgments and text comprehension performance were substantial and significant in both groups (Table 2).

In a first step, we compared recognition and monitoring resolution abilities between native and non-native speaking children in the paired-associates task. We conducted a multivariate ANOVA with recognition, monitoring discrimination scores, and Gamma correlations as dependent variables. Using Pillai’s trace, there was no significant group difference between native and non-native speaking students, *F*(3, 68) = 0.4; *p* = 0.75; ${\upeta }_{p}^{2}$ = 0.02. Thus, as hypothesized and replicating findings from Study 1, native and non-native speaking children did not differ significantly in their metacognitive monitoring (resolution) and recognition in the paired-associates task.

We compared monitoring resolution between native and non-native speaking children for the text comprehension task in a second step. We conducted a multivariate ANOVA with text comprehension, monitoring discrimination scores, and Gamma correlations as dependent variables. Using Pillai’s trace, there was a significant group difference between native and non-native speaking students, *F*(3, 68) = 4.20; *p* < 0.01; ${\upeta }_{p}^{2}$ = 0.16. We followed up the multivariate ANOVA, with separate univariate tests on the dependent variables (text comprehension, monitoring discrimination scores, and Gamma correlations). Univariate tests revealed that native speakers answered significantly more open-ended questions correctly than non-native speakers, *F*(1, 70) = 11.36; *p* < 0.01; ${\upeta }_{p}^{2}$ = 0.14. Interestingly, univariate tests revealed neither group differences on the monitoring discrimination score, *F*(1, 70) = 0.57; *p* = 0.45; ${\upeta }_{p}^{2}$ = 0.01, nor on Gamma correlations, *F*(1, 70) = 0.45; *p* = 51*.*; ${\upeta }_{p}^{2}$ = 0.01. In sum, native speaking children significantly outperformed non-native speaking children in terms of correctly answered questions. However, we did not find differences between native and non-native speakers in terms of monitoring resolution.

To gain further insights into how language competencies (teacher ratings) may explain performance and monitoring in native and non-native speakers, we computed non-parametric correlations. For native speakers, language competencies significantly correlated with performance in the text comprehension task (*r* = 0.63; *p* < 0.01). However, none of the other variables significantly correlated with language competencies. In other words, in both tasks (paired-associates and text comprehension), neither recognition nor monitoring resolution measures (discrimination scores and Gamma correlations) were related to language competencies in this subsample.

For non-native speakers, we found a different pattern of results. Language competences were significantly correlated with both performance (*r* = 0.47; *p* < 0.01), and Gamma correlations (*r* = 0.41; *p* < 0.05) in the text comprehension task. Furthermore, we found marginal correlations between language competences and monitoring discrimination scores in the paired-associates task (*r* = 0.30; *p* = 0.07), and in the text comprehension task (*r* = 0.30; *p* = 0.07). However, correlations between language abilities, recognition, and Gamma correlations in the paired-associates task were non-significant.

## General discussion

In Study 2, we investigated metacognitive monitoring (resolution) of native and non-native speaking 4^th^ graders in a paired-associates task (Kanjis) and a text comprehension task. The paired-associates task replicated the findings of Study 1. Native and non-native speaking students did not differ in recognition and metacognitive monitoring resolution measures (discrimination scores and Gamma correlations) in the paired-associates task. In Study 2, in addition to the Kanji task used in Study 1, we included a text comprehension task. In line with our expectations, native speaking subjects answered more open-ended questions correctly than non-native speaking participants. However, native and non-native speakers did not differ in metacognitive monitoring resolution measures (discrimination scores and Gamma correlations) in the text comprehension task.

We did not find recognition differences between native and non-native speakers in the paired-associates task, but native speaking children outperformed non-native speakers in the text comprehension task. Those results align with our expectations, based on findings that performance differences are most significant in language related tasks (OECD 2012). The included teacher ratings of language abilities confirmed that native speaking students had higher language abilities than non-native speaking students. This may be a relevant finding for future research investigating metacognition in children with various language backgrounds and abilities, as first-order task performance impacts metacognitive skills (Rinne and Mazzocco 2014; Roebers and Spiess 2017). Monitoring measures of native and non-native speakers may be more comparable in a paired-associates task than in a text comprehension task because first-order task performance is more similar in the paired-associates task than in the text comprehension task.

Most importantly, we did not find differences in metacognitive monitoring abilities (resolution) between native and non-native speaking students. Our results reveal that native and non-native speaking students do not differ in monitoring resolution and, hence, are both relatively well able to monitor their performance. Those findings contrast to research suggesting a multilingual advantage in higher order cognitions (Adesope et al. 2010; Grundy and Timmer 2017). Note that the multilingual advantage occurs when the multilinguals are assessed in their dominant language (Grundy and Timmer 2017). We assessed all participants in the language of instruction, which was, by definition, not the dominant language of the non-native speakers. Furthermore, contrary to typical multilinguals, non-native speakers in the present study were not proficient in the instruction’s language as native speakers. The bilingual advantage in higher order cognition may depend on language proficiency and the language of assessment. Therefore, it may be that non-native speakers would outperform their native speaking peers in monitoring abilities if (1) the non-native speakers would be multilingual (very proficient in more than one language) and/or (2) the non-native speakers would be assessed in their dominant language. In future research, it would be interesting to account for language proficiency and language of assessment to gain a more differentiated perspective of metacognitive monitoring in non-native speakers.

We followed up our analyses with correlations to gain more insight into the relationship between language competencies and metacognition. For the paired-associates task, our results reveal that the language abilities of non-native speakers are marginally associated with monitoring discrimination. However, we did not find any relations for the language abilities of native speakers in the paired-associates task. This finding suggests that monitoring and performance in the paired-associates task may be independent of one’s language abilities. In contrast, we found significant correlations between native and non-native speakers’ language abilities and performance in the text comprehension task. This is in line with research suggesting an impact of language abilities in language related tasks (OECD 2012). Furthermore, monitoring resolution measures (discrimination scores and Gama correlations) of text comprehension were related to language abilities for non-native speakers, but not for native speakers. Our findings indicate that the relation between language abilities and metacognitive monitoring may be task- (language-reduced vs. language-based) and participant- (native vs. non-native speaking) specific.

Our results are partly in line with studies suggesting a link between language abilities and declarative metacognition (Annevirta et al. 2007; Ebert 2015; Lecce et al. 2010; Lockl and Schneider 2007). On the one hand, the language abilities of non-native speakers seem to be related to monitoring resolution measures in a text comprehension task. On the other hand, the present research suggests that metacognitive monitoring resolution does not necessarily differ between native and non-native speakers and, thus, monitoring abilities do not seem to be strongly affected by language competencies. This may implicate that metacognitive monitoring skills are relatively independent of the native language and the language of assessment. Once a child understood the instructions, it can ask himself how confident it is about a particular item in any language. It might be that language abilities are more closely related to declarative aspects than procedural aspects of metacognition. As Ebert (2015, p. 562) stated: «The most important variable in shaping children’s knowledge about the mental world is probably language.» An interesting question for future research would be to clarify the role of language abilities for declarative and procedural aspects of metacognition.

Is it possible that the instruction language competencies are related to metacognitive abilities and thus explain performance differences? Language competencies seem to be related to metacognitive monitoring (resolution) for non-native speakers in a language related task (text comprehension). However, monitoring abilities do not seem to be generally impaired by this relationship, as indicated by similar monitoring resolution scores for native and non-native speakers. Metacognitive monitoring abilities may not be the primary source of performance differences between native and non-native speaking students. Still, metacognitive monitoring may be a valuable resource to address the performance gap among non-native speaking students. Accurate monitoring of one’s task performance is an essential precondition for implementing successful control strategies, such as allocating learning time to perceived item difficulty (Destan et al. 2014; Schneider and Lockl 2002; Schneider and Löffler 2016). This enables an individual to learn efficiently and improve one’s performance (Dunlosky and Metcalfe 2009). It would be interesting to assess metacognitive control processes in native and non-native speaking students in future research. This might contribute to further insights into metacognitive processes and how they are related to school performance of non-native speakers.

A strength of the present study is replicating the findings for Study 1 in a different sample in Study 2. We included a paired-associates and a text comprehension task, allowing us to take a distinguished perspective on monitoring differences between native and non-native speakers in different learning tasks. Furthermore, we made a first step connecting language abilities and procedural metacognition, a so far neglected topic in metacognition research. Despite the strengths, we need to acknowledge some limitations. We did not collect information about the socio economic status (SES) of the subjects. SES is a common confounding variable when addressing students’ language skills (cf. Glick and Clark 2012). We do not have detailed insights into how long children were used to following non-native language instructions. Therefore, it is challenging to account for individual differences in the non-native speaking group. We did not assess general cognitive abilities and could not control cognitive variables other than language when we matched the subjects. Children did not differ in performance in the paired-associates task, which may indicate similar cognitive abilities. Finally, our findings are limited to resolution measures of metacognitive monitoring in a paired-associates and a text comprehension task. To gain a more general perspective on monitoring abilities in native and non-native speakers, future research may include various measures of monitoring (e.g. resolution and calibration measures) in different cognitive tasks (e.g. recognition, text comprehension, free recall, perceptual tasks…).

## Conclusion

We assessed metacognitive monitoring resolution of native and non-native speaking 4^th^ graders in two similar yet independent samples. Our results are twofold. For one, we showed that native speaking students outperformed their non-native speaking peers in a language related task (text comprehension) but not in a language reduced learning task (picture based paired-associates). This is in accordance with previous research, indicating that performance differences may be more pronounced in language related tasks (OECD 2012). Most importantly, we did not find differences in metacognitive monitoring between native and non-native speaking children, independent of whether the task was language related or not. This suggests that metacognitive monitoring may not be the primary source of school performance differences between native and non-native speakers. Nevertheless, it might still be a valuable resource for non-native speaking students. Further research is needed to clarify the role of additional aspects of procedural metacognition in non-native speaking children’s school performance.

## Data Availability

Not applicable.
